# Peptides’ role in autism with emphasis on exorphins

**DOI:** 10.3402/mehd.v23i0.18958

**Published:** 2012-08-24

**Authors:** Karl L. Reichelt, Dag Tveiten, Anne-Mari Knivsberg, Gunnar Brønstad

**Affiliations:** 1 Department of Pediatric Research, Oslo University Hospital, Rikshospitalet, Oslo, Norway; 2 Biomedical Laboratory, Rådmann Halmrast, Sandvika, Norway; 3 National Centre for Reading Research, University of Stavanger, Stavanger, Norway; 4 Neurozym Pharma AS, Snåsa, Norway

**Keywords:** autism, peptides, exorphins, mass-spectroscopy

## Abstract

**Problem:**

The nature of the peptides found increased in urine from autism needs verification of their structure, especially those that show opioid activity.

**Methods:**

The peptides were separated on reverse phase C-18 HPLC in Trifluoroacetic acid–acetonitril gradients. Peaks eluting where synthetic opioids appear, and peaks that are common to most autistic children were analyzed by mass spectrometry and fragmentation pattern on a quadropole mass-spectrometer.

**Results:**

We could demonstrate exorphins in the urine from autistic children, and their length varied from one patient to the next.

**Conclusion:**

Exorphins are found in urine of autistic children and may account for their symptoms.

Like most medical and psychiatric diagnoses, autism is a symptomatic description of probable syndromes. Cardinal symptoms are an extreme introversion (aloofness), social indifference, stereotyped repetitive behaviours, language problems, problems with hygiene, temper tantrums and in some cases hyperactivity, irrational fears and decreased but fluctuating insensitivity to pain. Autism was shown to have a considerable genetic disposition (1, 2) which invariable must entail chemical changes. However, increasing rates point to a toxicological element since genetic drift is a very much slower process. Published data point to mercury (Hg) as a strong candidate (3–5).

**Table 1:** Some known exorphins are listed (one letter code)

Glumorphin	
A5	G-Y-Y-P-T; A4:G-Y-Y-P
B5	Y-G-G-W-L; B4:Y-G-G-W
C	Y-P-I-S-L
From alpha-casein	R-Y-L-G-Y-L-(E)
	Y-V-P-F-P (and amide)
From beta-casein	Y-P-F-P-G-P-I-P
Short forms down to	Y-P (amide)
Casorphin	Y-P-S-F (amide)
Alpha-lactorphin	Y-G-L-F (amide)
Beta-lactophin	Y-L-L-F (amide)
Gliadinomorphin	Y-P-Q-P-Q-P-F
β-Casomorphin (h)	Y-P-F-V-E-P-I

Note: The bovine form of casomorphins are shown. The human (h) is different in sequence.

An increase in urinary peptides has been found (6–9) and also an increase in opioid activity (6, 8–11). To be relevant to autism the following must apply:

1. The peptides must be there
2. Their nature elucidated
3. Bioactivities must be of relevance
4. Explain the antibodies found
5. A food behaviour axis must be possible
6. Removal of peptide source ought to give improvement

## Methods

Diagnosis of patients over many years was based on DSMIII and IV. The children discussed here have all been diagnosed by certified specialists working in several countries (Table 2). All were at the time without drugs and had been so for at least 6 months. The degree of handicap was rated by CARS (childhood autism rating scale). Various other tests were used in intervention studies (see later).

**Table 2:** Area under the 215 nm curve eluting after hippuric acid. Arbitrary units

	Italy	Serbia	Slovenia	Control
Mean	403	584	564	289
SD	307	504	523	84
*N*	145	139	51	181
95% CI	343	500	416	277
	453	667	711	302
*P*	<0.0001	<0.0001	0.0005	

The prepubertal children were picked out and their age ranged from 2 to 13 years. First morning urine was collected and frozen as soon as possible to −15°C. The urine samples were left to thaw over night in the laboratory cold room (4°C) and creatinin was measured by the Department of Clinical Biochemistry, Rikshospitalet, Norway. Eight-hundred microliters were centrifuged through Spin-X Centrifuge Tube filters from Costar at 3000×g for 20 min at room temperature. The tubes were polypropylene and the filter 0.22 µm Cellulose acetate.

An aliquot equivalent to 250 nanomoles creatinin was applied to reverse phase HPLC columns (Vydac c-18 Peptide/protein columns cat 218TP54 from USA). The columns were eluted with TFA (trifluoroacetic acid) 10 mM and acetonitril gradients as described (11). Elution was monitored at 215 nm (peptide bonds), 280 nm (aromatic groups) and 325 nm (indolyl groups). Rate of elution was 1 ml/min and the gradient was started after a 15 min isocratic TFA run.

To determine which peptide peaks should be studied since considerable variation was found both in quantity and properties of eluted peaks, urine from five to ten reasonably similar autistic urines were combined and rerun. Peaks that are common would stay the same or increase, while individual peaks are diluted out. (This technique was learned from P Shattock OBE, Sunderland, UK.) The relevant peaks were lyophilized and stored at −15°C until used further. Peaks that elute co-chromatographically with synthetic standards were also collected off line.

## Mass spectroscopy

Lyophilised material from collective runs were re-dissolved in methanol/water (1/1) and 10 mM formic acid and 300 µl of this buffer. After stirring at room temperature this was applied to a P-sciex mass spectrometer by micropump and direct inlet. Prominent ion peaks were fragmented by MS/MS. Each peptide had to have declustering potential and collision energy adjusted for optimal results.

Using Elisa technique anti food protein antibodies were analyzed in serum commercially by Fürsts Laboratory in Oslo, while recently we have been using the research laboratory at the University of Bergen Hospital (Haukeland).

As for ethical considerations, none of our procedures are damaging to the patients.

Late onset and more regressive autism has statistically increased peptide levels compared to early onset autism. Late onset 1520 units±926 units *n*=25, neonatal onset 570±188 (*n*=45) and *p*=0.0001 Mann Whitney *U*-test, two-tailed.

The urines were obtained in Italy by Association Planet Autism; from Serbia by Dr Selakovic, Beograd; from Slovenia by D. Patterson, MD, of Runaway Bay, Queensland, Australia. Controls are Norwegian children provided by education specialist M. Nödland, Stavanger. The age range was 3–12 years for controls, for patients 2–13 years. The level for Scandinavian children has been published previously (6, 11). High functioning autistic children overlap with controls to a degree which makes urine diagnosis difficult (13).

## Results

**Fig. 1:**
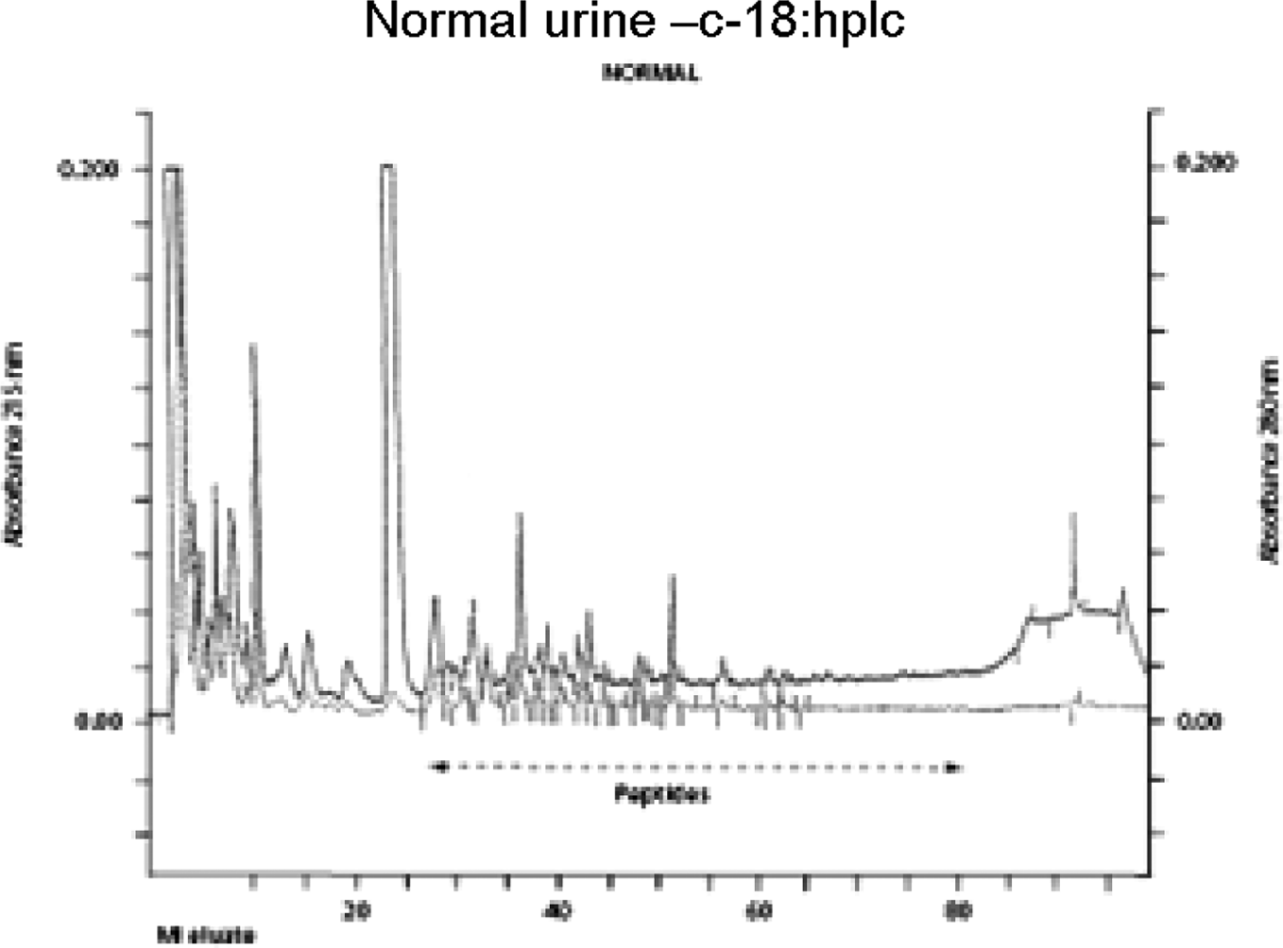
The HPLC pattern of a normal urine equivalent to 250 nanomoles creatinin.

**Fig. 2:**
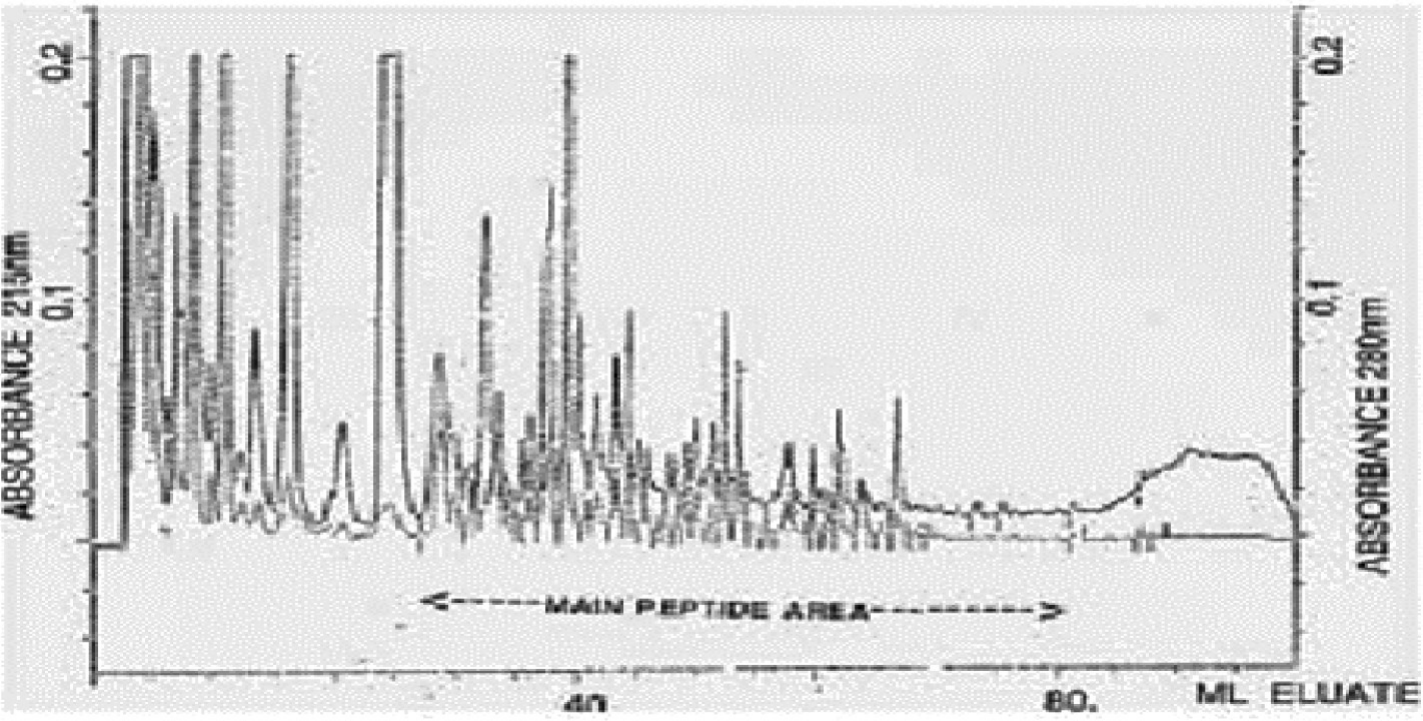
The HPLC pattern of a boy with infantile autism.

**Fig. 3:**
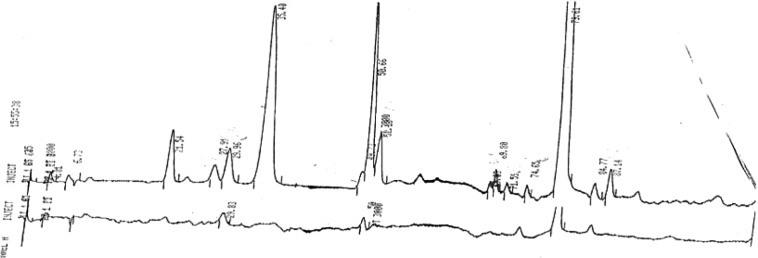
Bottom trace amino acid analysis before hydrolysis, and top trace after hydrolysis in 6 M HCl in closed vials at 110°C for 16 hours. The late eluting (to the right) large peak is ammonia.

**Fig. 4:**
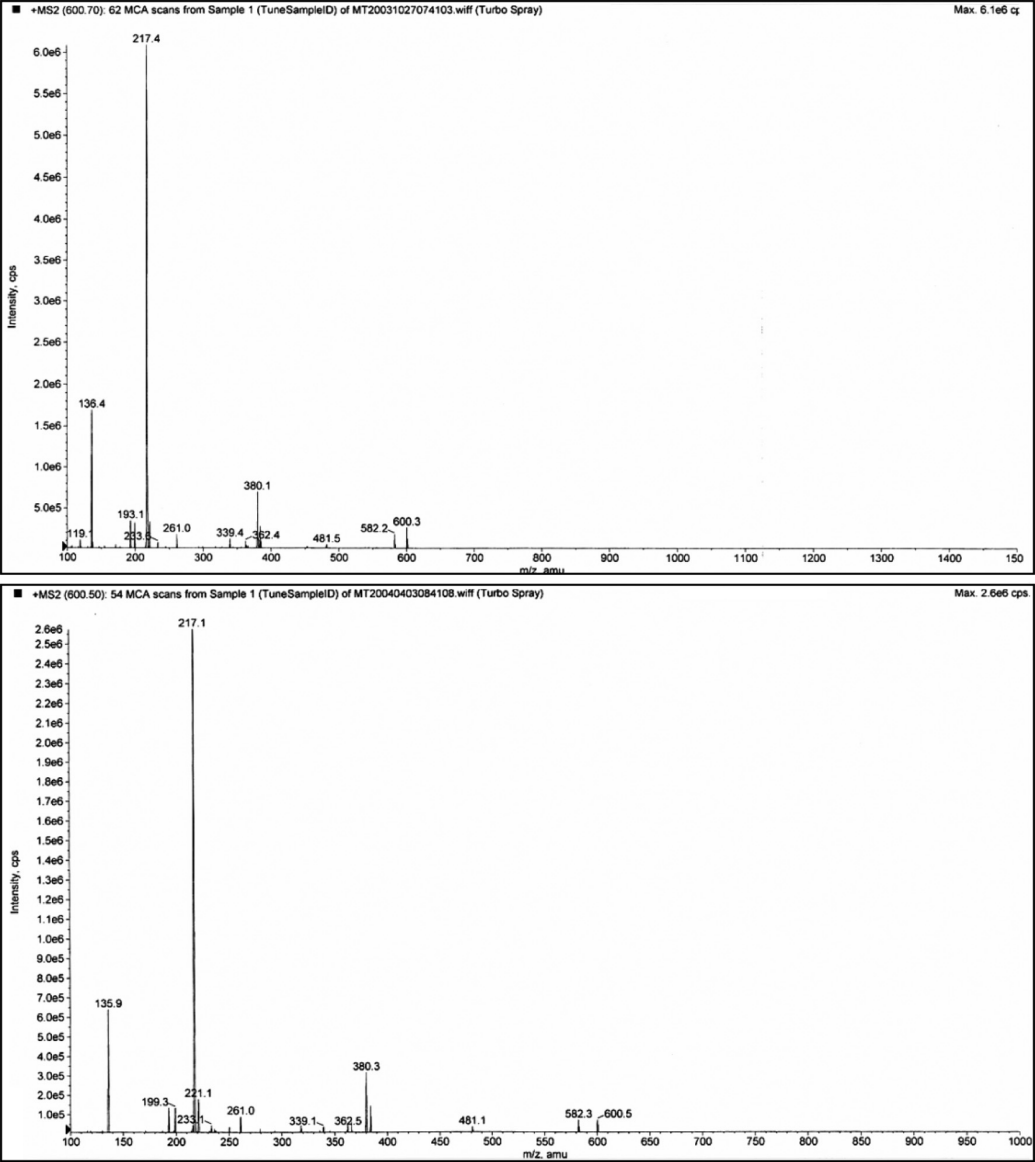
Demonstration of glumorphin A-5 by MS/MS (fragmentation). Top trace is synthetic standard, and bottom trace Glumorphin A5 isolated from regressive autistic children's urine. Declustering potential was set at 20 and collision energy at 30. The X axis has been extended in bottom trace to secure against hidden ion peaks.

**Fig. 5:**
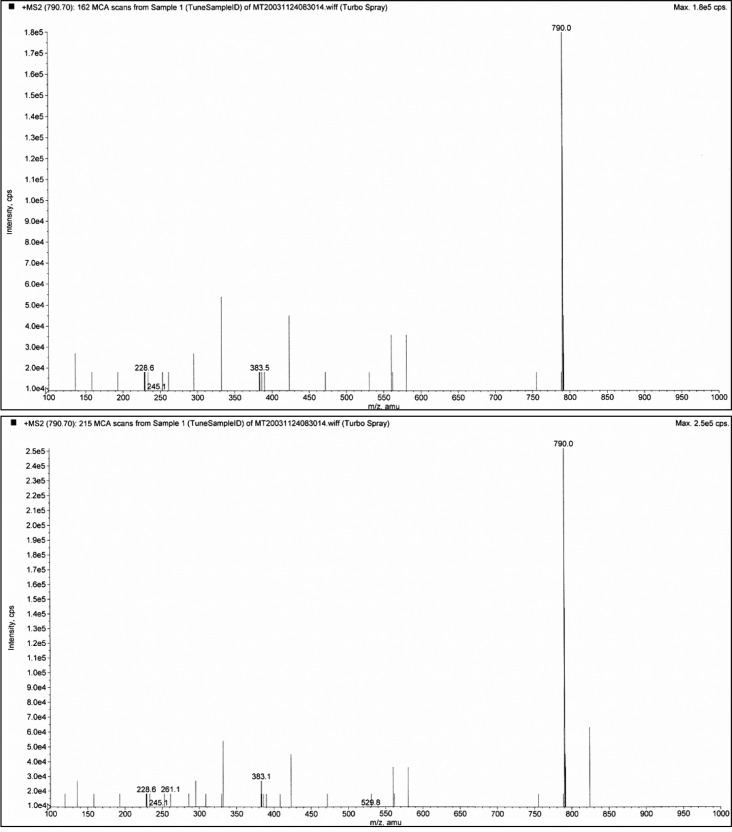
Casomorphin 1–7. The casomorphin 1–7 standard is the top trace, from CPDD bottom trace. The peak appearing after the intact ion could be removed by increasing the declustering potential. It is shown here to illustrate the strong propensity for peptides to form aggregates and adducts. Declustering here is 20 and collision potential is 70.

Typical normal and infantile autistic HPLC patterns are seen in Fig. 1 and Fig. 2. In Fig. 3 we show peaks from the HPLC run where hydrolyzed amino acid analysis is compared with a not hydrolyzed equal aliquot. Clearly a rather massive increase in amino acids demonstrate that most of the peaks eluting after hippuric acid are peptides. In Fig. 4 the fragmentation pattern of Glumorphin A5 prove its presence in CPDD children. Likewise in Fig. 5 β-casomorphin 1–7 is proved present, but is found in only some of the autistic spectrum children. (perhaps 5–10%). Other casomorphins are often found too (see appendix), and more frequently. Finally we can demonstrate that overloading a normal person with gluten and gliadin for three days resulted in finding Glumorphin B5 in urine. The peptide was determined to four decimal places and showed identity through spiking with synthetic glumorphin B5.

**Fig. 6:**
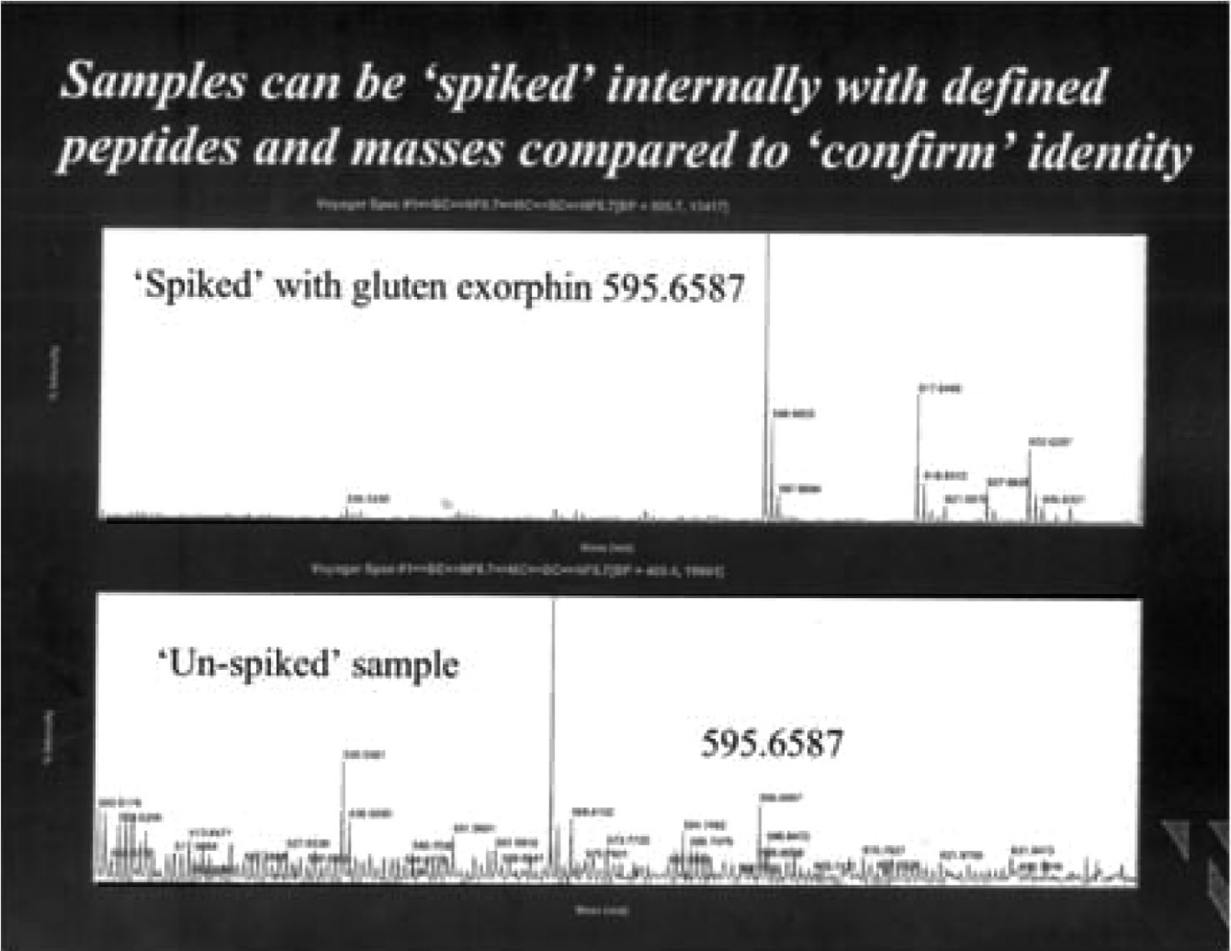
Overloading normal person with gluten and gliadin for three days resulted in a demonstrable glumorphin B 5. Topp trace is spiked with standard peptide, bottom trace urine pattern after three days excessive gluten exposure.

## Discussion

Some of the peptides found are bio-active. Thus opioid receptor binding (14) and analgesia studied by the tail flick test (15) have also been found. Furthermore, IV injection caused hyperactivity (15, 16) and Fos antigen activation in key nuclei of rat brain (17), such as the nucleus accumbens. Both analgesia and Fos antigen increase were blocked by naloxone. The opioid peptide also caused decreased uptake of dopamine in striatal synaptosomes and showed *in vivo* dopaminergic hyperactivity using the Ungerstedt model (15).

Also linking autism to dietary proteins was the discovery of antibodies against gluten, gliadin and casein increased in autistic children (8, 18, 19–21).

A Russian group (10) found that casomorphin in the blood was inversely related to psychomotor development in infants. Casomorphins have, furthermore, been shown to abrogate separation distress call and reaction in newly hatched chickens (22) and also causing social isolation in kittens and pups.

## A gut to brain interaction exists

Peptides are formed in the gut from food proteins (23, 24) and everyone takes up peptides from the gut (24, 25). If peptidases are inhibited or lacking, this uptake is increased (26, 27). Opioid peptides also get across the blood–brain barrier (28, 29).

Antibodies against food proteins increased in autism points to increased uptake of protein. Humans take up intact protein (25, 30) and intact enzymes (31). These proteins can be recovered from mother's milk (32, 33). Botulinum toxin is a peptidase that is taken up from the gut, crosses the blood–brain barrier and the pre-synaptic membrane and splits one peptide bond in SNAP-25 with fatal results (34). Some antibodies, for instance against gliadin (35), have direct effects on Purkinje cells of the cerebellum. Considerable intestinal problems are found in autistic spectrum disorders (36–38) which would result in increased uptake. Casein and gluten can also release inflammatory cytokines in autism (39) and such cytokines increase permeability of endothelial barriers (40). In autism increased gut permeability has been established (41, 42) and, therefore, the prerequisite conditions for the postulated mechanism are present.

Given the established data relating certain food proteins to autism it would be advisable to remove these from the diet. This has been done and quite a number of such studies have been conducted (6, 7, 43–52). The length of the intervention is critical and short term trials such as 14 days are quite inadequate. Double blind dietary studies are very demanding, but single blind pair wise matched and randomly assigned studies (50), as well as testing over 4 years to counter act placebo (51), are promising, and indicate that dietary intervention is effective.

In Table 3 we summarize the data from an open 4-year follow up and in Table 4 the results are shown of matched randomly assigned children with autism. Note that in the 4-year study children that quit the GF/CF diet, the children regressed (0.58). The numbers are small, but since diagnosis is symptomatic not aetiological, pair wise matched randomly assigned studies are much more secure because this increases the probability of comparing similar patients.

**Table 3:** Year open study of dietary intervention in 15 patients

Test	Initial score	1-year change	4-year gain	No	*p*-Value
C-Raven	6.8±2.8	+8.6±2.8	+8.6±3.2	12	0.005
ITPA	25.7±5.5	+2.7±2.5	+6.1±2.8	10	0.005
DIPAB test					
Social isolation	8.5±3.3	−6.1±2.7		14	0.005
Bizarre traits	5.3±2.2	−5.3±1.22		14	0.005

DIPAB, Diagnosticering af psykotisk Adfærd hos barn (Diagnosis of Psychotic behaviour in children); ITPA, Illinois test of psycholinguistic ability.ITPA, Illinois test of psycholinguistic ability.

The scores are the mean±standard deviation. Mann–Whitney *U*-test, two-tailed used throughout.

Five children had to be dropped from the ITPA test since the test has age limitations, and they were too old after 4 years. One of the 15 only participated for 6 months when he was convinced to quit by his GP.

**Table 4:** GF/Cf diet with control group for pair wise matched and randomly assigned autistic children (52)

Group	Diet	Diet	Control	Control	Diet compared with controls
Period	Start	12 m	Start	12 m	
Mean	12.5	5.6	11.5	11.2	
SD	2.2	2.4	3.9	5.0	
Significance		0.005*		0.798	0.001

Diet group *N*=10 and control group also 10. Mann Whitney U test two tailed was used.

Overloading normal person with gluten/and gliadin for 3 days gave a measurable peak for glumorphin B5, which could be spiked with B5 (Fig. 5). The peak is determined to four decimal places by mass spectroscopy. It is therefore possible that even with a normal genome but decreased expression number due to lower operator gene activity, insufficient break down capacity may be present in autistic children.

## Appendix 1

To ensure safer diagnoses a mass spectrometric fragmentation technique is being developed. In the figure below this is demonstrated for five collected autistic spectrum urines and synthetic standard for casomorphin 1–4. Dr K. Elgøen picked out the two best recognition peaks in the fragmentation pattern. A HPLC peak is even if similar, a like peak, but not necessarily the same as the spiked compound.

As can be seen the urine and the synthetic sample are the same. The autistc children were diagnosed by Prof A. Persico, The Open University, Rome, Italy and is cordially thanked for this help.

The figure demonstrates qualification ions from standard casomorphin (bovine) 1–4 in the top trace. In the lower trace the same peptide is shown from pooled autistic patients analyzed in a sample equivalent to 250 nanomoles creatinin. They are clearly the same.
